# Epidemiology and outcomes for level 1 and 2 traumas during the first wave of COVID19 in a Canadian centre

**DOI:** 10.1038/s41598-022-23625-8

**Published:** 2022-11-27

**Authors:** S. Boutin, J. Elder, N. Sothilingam, P. Davis, T. Oyedokun

**Affiliations:** 1 Department of Emergency Medicine, Royal University Hospital, University of Saskatchewan, 103 Hospital Drive, Saskatoon, SK S7N 0W8 Canada; 2 Department of Surgery, Royal University Hospital, University of Saskatchewan, 103 Hospital Drive, Saskatoon, SK S7N 0W8 Canada

**Keywords:** Health care, Medical research

## Abstract

To determine if lockdown measures imposed during the first wave of the COVID19 pandemic affected trauma patterns, volumes, and outcomes in a western Canadian level 1 trauma center, we performed a retrospective cohort study assessing level 1 and 2 trauma patients presenting to our center during the initial COVID19 “lockdown” period (March 15–June 14, 2020) compared to a similar cohort of patients presenting during a “control” period 1 year prior (March 15–June 14, 2019). Overall, we saw a 7.8% reduction in trauma volumes during the lockdown period, and this was associated with a shorter average ED length of stay (6.2 ± 4.7 h vs. 9.7 ± 11.8 h, p = 0.003), reduced time to computed tomography (88.5 ± 68.2 min vs. 105.1 ± 65.5 min, p < 0.001), a reduction in intensive care unit admissions (11.0 ± 4.9% vs. 20.0 ± 15.5%, p = 0.001), and higher injury severity score (6.5 ± 7.6 vs. 6.2 ± 9.5, p = 0.04). Our findings suggest that lockdown measures imposed during the first wave of the COVID19 pandemic had a significant impact on trauma patients.

## Introduction

With the rapid spread of COVID19 across the world, public health measures such as stay-at-home orders and physical distancing were imposed to reduce virus transmission and virus-related hospital admissions^1^. Additionally, several hospitals adapted the layout of trauma bays to reduce the possibility of fomite contamination by viral particles, including placing all non-essential supplies outside the trauma bay^2^. Many trauma centers have also deemed all trauma patients to be COVID19 positive until proven otherwise, requiring the donning of appropriate personal protective equipment (PPE) before contact with these patients^1,3–6^. The healthcare effects of these public health measures have been variable; in some cities, traumas and admissions due to trauma decreased, effectively reducing strain on the healthcare system at the beginning of the pandemic^7–13^. Furthermore, the pattern of trauma activations has shown a trend towards increased non-accidental penetrating trauma and a reduction in blunt trauma due to motor vehicle collisions^14–18^. In hospitals, it appears the pandemic may have improved efficiency, with many centers reporting reduced lengths of stay and reduced times to surgery compared to pre-pandemic times^19,20^.

Our experience suggests that most measures implemented to reduce the spread of COVID19 in the hospital setting, such as the use of appropriate PPE, require a trade-off between time to patient treatment and HCW protection. Specific measures to reduce the viral spread in our center included the creation of designated “intubation teams” responsible for airway management, endotracheal intubation, and arterial and central venous line placement. We also implemented the use of an intercom to facilitate communication between members of the team inside the trauma bay and those outside, separated by glass doors. Additionally, direct physical access to the radiologist reading room adjacent to the trauma bay was eliminated.

To our knowledge, there has been no published study from western Canada assessing how measures implemented to reduce the spread of COVID19 have affected trauma care, especially in a predominantly rural population. Saskatchewan has one of Canada’s lowest provincial population densities and a relatively high proportion of rural-based citizens^21^, making our center unique in that we often see trauma patients who have been transported over long distances prior to assessment. This study aimed to determine if measures implemented to curtail the spread of COVID19 had any impact on the nature and outcome of levels one and two trauma patients presenting to our western Canadian urban level one trauma center. We hypothesized that during the COVID19 pandemic, the rate of level one and two trauma activations would be less, but outcomes would remain unchanged.

### Study design and time period

After obtaining ethics approval from the University of Saskatchewan Biomedical Research Ethics Board and the Saskatchewan Health Authority Research Ethics Board (Bio REB-2322), we identified a historical cohort of patients presenting to our level one trauma center (Royal University Hospital, Saskatoon SK) during the initial COVID19 “lockdown” period (March 15–June 14, 2020). For comparison, a similar cohort of patients presenting to our level 1 trauma centre during a “control” period one year prior was also identified (March 15–June 14, 2019).

#### Study setting

The Royal University Hospital is one of the two trauma centers in Saskatchewan and serves the city of Saskatoon, surrounding communities, and the rural and remote communities of northern Saskatchewan. Data was collected from the trauma database through our Digital Health Analytics unit and from electronic records obtained from Sunrise Clinical Manager (SCM) at the RUH health records department.

#### Patient population

Patients were included if they were triaged as a level 1 or 2 trauma as per our local trauma criteria (see Supplementary Fig. 1) during the study period. Charts were reviewed electronically using a piloted data collection form. Variables of interest included age, gender, comorbidities, mode of arrival, mechanism of injury (i.e. blunt or penetrating), blood alcohol level, time to computed tomography (CT, defined as arrival time to CT), time to electrocardiogram (EKG, defined as arrival time to EKG), emergency department (ED) boarding time (defined as arrival time to admission to an inpatient bed), the need for intubation and mechanical ventilation, and 30-day mortality. The Charlson Comorbidity Index (CCI) and injury severity score (ISS) were also calculated based on data obtained from a manual chart review.

#### Outcome measures

The primary outcome of interest was the number of level 1 and 2 trauma activations during the control and lockdown periods. Secondary outcomes of interest included time to CT, time to EKG, the proportion of hospital admissions, ICU admissions, hospital length of stay (LOS), and 30-day mortality. During trauma activations in our center, we use EKG as an adjunct in the workup of trauma patients to detect arrhythmias, signs of blunt cardiac injury, and potential underlying cause for the trauma. Our EKG technicians are paged with the activation of our trauma team, so we included time to EKG as a surrogate measure of efficiency.

All data collection, analysis, and storage were completed in compliance with the University of Saskatchewan Research Ethics Board guidelines. The need for direct consent for data collection from subjects was waived by the University of Saskatchewan Biomedical Research Ethics Board, as participants were not personally identified, had already been medically treated, and were not contacted in relation to this research. Additionally, participants’ medical care was not impacted by this research, their personal information will not be disclosed, and they will not be asked to recount their experience or trauma.

#### Data analysis

We used the *T* test and Wilcoxon non-parametric test to compare normally and non-normally distributed continuous variables, respectively, between groups (2019 vs. 2020). Categorical variables were compared using the Chi-square test and Fisher’s exact test as appropriate. All datasets generated during this study are available in Supplementary Material 2.

## Results

During the lockdown period, we identified 224 trauma activations compared to 243 trauma activations presenting to our center during the control period. Of note, our ED volumes were significantly lower during the lockdown compared to the control period (12,045 vs. 16,319 visits, respectively). Taken together, this suggests an increase in the proportion of trauma activations presenting to our center (1.86 vs. 1.48% of ED visits).

The characteristics of trauma patients presenting to our center are shown in Table 1. Overall, there were no differences in patient age, gender, or comorbidities between the control and lock-down periods.

**Table 1:** Patient characteristics of trauma activations during the control and lockdown period.

Patient factor	Control period (N = 243)	Lockdown period (N = 224)	p value
Age (mean, SD)	36.2 (18.7)	34.7 (18.2)	0.43
Pediatric (N, %)	10.3 (25)	10.3 (23)	0.99
Male (N, %)	169 (69.5)	167 (74.5)	0.23
Charlson Comorbidity Score (mean, SD)	0.61 (1.21)	0.73 (1.58)	0.65

*SD* standard deviation.

*Pediatric age was considered any patient under 17 years old.

The characteristics of trauma activations during the control and lockdown periods are shown in Table 1. Overall, there was a slight increase in the overall ISS during the lockdown period (6.51 vs. 6.15: p = 0.04).

Outcomes of trauma activations during the control and lock-down period are highlighted in Table 3. Overall, time to CT was improved (88.5 vs. 105.1 min: p < 0.001), as was overall ED LOS (6.2 vs. 9.7 h; p = 0.003). Lastly, we noted a decrease in the proportion of patients admitted to ICU (11.0 vs. 20.0%; p = 0.001).

## Discussion

### Interpretation of findings

The COVID19 pandemic has forced healthcare systems to re-evaluate their resource distribution and standards of practice. For example, many centers have adopted new trauma protocols in response to the COVID19 pandemic to protect healthcare workers from possible COVID19 transmission. In our center, screening criteria for patients presenting to the ED were introduced, donning of additional personal protective equipment (PPE) was required, and additional sanitation protocols were introduced for all trauma patients. Patients unable to respond to screening questions, including many trauma patients, were automatically considered positive for COVID19. We hypothesized that introducing these new protocols would increase the time to investigative treatment and ED length of stay due to the increased time required to don PPE. However, our data suggest that resource allocation may have become more efficient during the pandemic, with patients experiencing shorter ED lengths of stay, reduced ICU admissions, and shorter time to CT imaging (Table 3). At the same time, the injury severity of level 1 and 2 trauma patients increased during the pandemic (Table 2), and the overall proportion of level 1 and 2 trauma patients also increased (from 1.49 to 1.86% of total ED visits). Hospital length of stay, demographics of presenting patients, number of patients intubated, time to X-ray/EKG, and 30-day mortality were not statistically different among analyzed groups (Table 1; Table 3).

**Table 2:** Trauma characteristics of Trauma Activations during the control and lockdown period.

Trauma factor	Control period (N = 243)	Lockdown period (N = 224)	p value
Level 1 trauma (N, %)	113 (47.3)	111 (49.6)	0.63
Penetrating trauma (N, %)	51 (21.1)	62 (27.7)	0.1
**Injury severity score (mean, SD)**	**6.2 (9.5)**	**6.5 (7.6)**	**0.04**
Suicide attempts (N, %)	12 (5.1)	18 (8.0)	0.2
ETOH intoxication (N, %)	47 (38.5)	43 (42.6)	0.53

Significant values are in bold.

*EtOH* alcohol, *SD* standard deviation.

**Table 3:** Trauma Outcomes of trauma activations during the control and lockdown period.

Trauma outcome	Control period (N = 243)	Lockdown period (N = 224)	p value
*Time to X-ray (mins; mean, SD)*	115.5 (154)	104.6 (121)	0.77
*Time to EKG (mins; mean, SD)*	85.8 (108)	64.4 (68.7)	0.09
***Time to CT (mins; mean, SD)***	**105.1 (65.5)**	**88.5 (68.2)**	** < 0.001**
*intubated (N, %)*	20 (8.2)	23 (10.3)	0.44
*Admitted (N, %)*	130 (53.5)	111 (49.6)	0.39
***Admitted to ICU (N, %)***	**20 (15.5)**	**11 (4.9)**	**0.001**
***Average ED LOS (hours; mean, SD)***	**9.7 (11.8)**	**6.2 (4.7)**	**0.003**
*Average hospital LOS (days; mean, SD)*	7.0 (33.3)	5.7 (19.3)	0.55
*30-day mortality (N, %)*	5 (2.1)	12 (5.4)	0.06

Significant values are in bold.

*EKG* electrocardiogram, *CT* computed tomography, *ICU* intensive care unit, *LOS* length of stay, *SD* standard deviation.

The reduced ED length of stay and time to CT imaging is perhaps explained by an overall reduction in ED visits seen during the first wave of the pandemic (16,319 vs 12,045, respectively). This reduction has similarly been reported in other ED’s within North America and Europe^13,22^. In addition, a reduction in departmental resource utilization (for example, decrease in ED CT usage for non-trauma-related patients) could have allowed trauma patients to take priority, reducing overall workup and disposition time from the ED. Additionally, our pre-alert trauma activation system potentially negated the impact of additional PPE requirements, with resources and staff mobilised prior to patient arrival. The reduction in ICU admissions may be explained by the possible prioritization of COVID19 patients by the ICU, the overall reduction in trauma numbers, and the reduction in ISS observed during the pandemic.

### Comparison to previous studies

To the best of the authors' knowledge, no studies have yet examined the impact of the pandemic on time to specific investigations (e.g. CT/EKG/X-ray) within the ED. However, multiple studies have demonstrated the impact of the COVID19 pandemic on patient presentations to the ED, acuity of presentations, and ED length of stay within North America. In concordance with the presented data, Baugh et al.^22^ demonstrated a decrease in ED patient visits throughout the pandemic in the United States (US), while the average acuity of patient presentations increased. Additionally, Besoff et al*.* (2021) found a reduction in the number of pediatric trauma activations after introducing the first stay-in-place orders in the US, but an increase in the severity of pediatric traumas. Furthermore, several US centers reported an overall reduction in hospital length of stay and time to surgery early in the pandemic^19,20^. Also similar to our findings, Moyer et al*.* (2021) found a significant reduction in trauma admissions with no change in in-hospital mortality for trauma patients admitted before and during the first wave of the pandemic. Conversely, nationwide studies in the US demonstrate an increase in ED length of stay during the pandemic despite reduced overall ED volumes, but this data was not trauma-specific^23,24^. Interestingly, several US and European studies have shown an increase in non-accidental trauma, penetrating trauma^25^, abusive head trauma, and pediatric abusive trauma during the pandemic, highlighting an area of future research for our center^14,26–28^.

### Strengths

This study included patients from a large catchment area with well-defined patient data, examining specifically level 1 and level 2 traumas. This permitted direct analysis of the pandemic’s impact on local resources when delivering care to trauma patients. A standardized method of data collection was used to eliminate data entry variation^29^. Furthermore, comparing timeframes across the same period pre and during the pandemic eliminates seasonality bias in patient presentations. Finally, the detailed breakdown of services provides valuable information for subsequent analyses and future literature searches by other researchers.

### Limitations

There are several limitations to our study. One such limitation is that this was a retrospective chart review. Additionally, we included patients who had been transferred within Saskatchewan, all treated within the same health network, city, and at a single center; therefore, our data may have been impacted by regional practices. Furthermore, our observed data set was obtained during the early stages of the COVID19 pandemic within Saskatchewan. The observed impact of the pandemic is likely to fluctuate depending on the local COVID19 burden, which has increased substantially since the first wave of the pandemic. Future studies could evaluate the impact of the COVID19 pandemic on trauma care delivery during different phases of the pandemic to determine if the same findings would be noted when overall ED volumes increase.

### Clinical and research implications

Our findings suggest the public health measures imposed to curb the spread of COVID19 have resulted in a reduction in level 1 and 2 traumas but an increase in trauma severity, underscoring the importance of community awareness and education programs aimed at reducing both blunt and penetrating traumas. Additionally, the reduction in ED length of stay, time to CT, and reduction in ICU admissions suggests that rapid assessment and workup of trauma patients may improve their short-term outcomes. Further research assessing the exact type of trauma seen during the pandemic in various subgroups, including pediatrics, could help guide resource allocation and call schedules to ensure patients have rapid access to specialist trauma surgeons. Additionally, investigating how access to resuscitation products such as blood, tranexamic acid, and other interventions during the initial trauma resuscitation period (e.g. the 1st hour after arrival in the ED) could provide further insight into the reduction in ICU admissions seen in our study despite an increase in injury severity. Our study serves as an initial step towards understanding how the pandemic has affected trauma activations and outcomes in a province with initially relatively low COVID19 cases. As the pandemic has evolved, our numbers have increased significantly, and future studies should identify how an increased overall ED volume has affected trauma activations and outcomes.

## Conclusions

In this retrospective chart review, we observed that the COVID19 pandemic impacted resource utilization when providing trauma care within our local healthcare system, decreasing time to specific investigations, reducing ED length of stay, and reducing ICU admissions. As the pandemic evolves, its impact on local organizations will continue to change, particularly as responses shift from acute to chronic. Our findings suggest that new protocols introduced during the COVID19 pandemic may not have the anticipated effects on patient care, and emergency medicine organizations should be encouraged to continually assess the pandemic’s impact on their local resource utilization to help better adapt their local health system planning.

## Supplementary Information

Supplementary Information.

Supplementary Legends.

Supplementary Figure 1.

## Data Availability

Datasets generated during this study are available in the supplementary information.

## References

[1] The Committee on Trauma. Maintaining trauma center access and care during the covid-19 pandemic: guidance document for trauma medical directors. (2020).

[2] Livingston DH, Bonne S, Morello C, Fox A (2020). Optimizing the trauma resuscitation bay during the covid-19 pandemic. Trauma Surg. Acute Care Open.

[3] Christian MD, Sprung CL, King MA (2014). Triage: Care of the critically ill and injured during pandemics and disasters: CHEST consensus statement. Chest.

[4] Coleman JR, Burlew CC, Platnick KB (2020). Maintaining trauma care access during the COVID-19 pandemic: An urban, level-1 trauma center’s experience. Ann. Surg..

[5] Li Y, Zeng L, Li Z (2020). Emergency trauma care during the outbreak of corona virus disease 2019 (COVID-19) in China. World J. Emerg. Surg..

[6] Pieracci FM, Burlew CC, Spain D (2020). Tube thoracostomy during the COVID-19 pandemic: Guidance and recommendations from the AAST Acute Care Surgery and Critical Care Committees. Trauma Surg. Acute Care Open.

[7] Hernigou J, Morel X, Callewier A, Bath O, Hernigou P (2020). Staying home during “COVID-19” decreased fractures, but trauma did not quarantine in one hundred and twelve adults and twenty eight children and the “tsunami of recommendations” could not lockdown twelve elective operations. Int. Orthop..

[8] Comelli I, Scioscioli F, Cervellin G (2020). Impact of the covid-19 epidemic on census, organization and activity of a large urban emergency department. Acta Biomed..

[9] Forrester JD, Liou R, Knowlton LM, Jou RM, Spain DA (2020). Impact of shelter-in-place order for COVID-19 on trauma activations: Santa Clara County, California, March 2020. Trauma Surg. Acute Care Open.

[10] Park C, Sugand K, Nathwani D, Bhattacharya R, Sarraf KM (2020). Impact of the COVID-19 pandemic on orthopedic trauma workload in a London level 1 trauma center: the “golden month”: The COVid Emergency Related Trauma and orthopaedics (COVERT) Collaborative. Acta Orthop..

[11] Shi Y, Kvasnovsky C, Khan S (2021). Impact of the COVID-19 pandemic on trauma activations at a pediatric level 1 trauma center in New York. Pediatr. Surg. Int..

[12] Stoker S, McDaniel D, Crean T (2020). Effect of shelter-in-place orders and the COVID-19 pandemic on orthopaedic trauma at a community level II trauma center. J. Orthop. Trauma.

[13] Moyer JD, James A, Gakuba C (2021). Impact of the SARS-COV-2 outbreak on epidemiology and management of major traumain France: A registry-based study (the COVITRAUMA study). Scand. J. Trauma Resusc. Emerg. Med..

[14] Sherman WF, Khadra HS, Kale NN, Wu VJ, Gladden PB, Lee OC (2021). How did the number and type of injuries in patients presenting to a regional level I trauma center change during the COVID-19 pandemic with a stay-at-home order?. Clin. Orthop. Relat. Res..

[15] Sutherland M, McKenney M, Elkbuli A (2021). Gun violence during COVID-19 pandemic: Paradoxical trends in New York City, Chicago, Los Angeles and Baltimore. Am. J. Emerg. Med..

[16] Bessoff KE, Han RW, Cho M (2021). Epidemiology of pediatric trauma during the COVID-19 pandemic shelter in place. Surg. Open Sci..

[17] Devarakonda AK, Wehrle CJ, Chibane FL, Drevets PD, Fox ED, Lawson AG (2021). The effects of the COVID-19 pandemic on trauma presentations in a level one trauma center. Am. Surg..

[18] Pelzl CE, Salottolo K, Banton K (2021). COVID-19 and trauma: How social distancing orders altered the patient population using trauma services during the 2020 pandemic. Trauma Surg. Acute Care Open.

[19] Park C, Sugand K, Aframian A (2021). Impact of COVID-19 pandemic on hip fractures: The central London experience COVID-related urgent geriatric hip trauma (COUGH) study COVERT (COVid Emergency-Related Trauma and orthopaedics) collaborative. Ir. J. Med. Sci..

[20] Leichtle SW, Rodas EB, Procter L, Bennett J, Schrader R, Aboutanos MB (2020). The influence of a statewide “Stay-at-Home” order on trauma volume and patterns at a level 1 trauma center in the united states. Injury.

[21] Statistics Canada. Division No. 18, CDR [Census division], Saskatchewan and Saskatchewan [Province] (table). Census Profile. 2016 Census. Statistics Canada Catalogue no. 98–316-X2016001. Ottawa. https://www12.statcan.gc.ca/census-recensement/2016/dp-pd/prof/details/page.cfm?Lang=E&Geo1=FED&Code1=35078&Geo2=PR&Code2=35&SearchText=Ottawa--Vanier&SearchType=Begins&SearchPR=01&B1=All&GeoLevel=PR&GeoCode=35078&TABID=1&type=0 (Accessed 29 Nov 2017).

[22] Baugh JJ, White BA, McEvoy D (2021). The cases not seen: Patterns of emergency department visits and procedures in the era of COVID-19. Am. J. Emerg. Med..

[23] Boserup B, McKenney M, Elkbuli A (2020). The impact of the COVID-19 pandemic on emergency department visits and patient safety in the United States. Am. J. Emerg. Med..

[24] Lucero A, Sokol K, Hyun J (2021). Worsening of emergency department length of stay during the COVID-19 pandemic. J. Am. Coll. Emerg. Physicians Open.

[25] Waseem S, Nayar SK, Hull P (2021). The global burden of trauma during the COVID-19 pandemic: A scoping review. J. Clin. Orthop. Trauma.

[26] Staunton P, Gibbons JP, Keogh P, Curtin P, Cashman JP, O’Byrne JM (2021). Regional trauma patterns during the COVID-19 pandemic. Surgeon.

[27] Sidpra J, Abomeli D, Hameed B, Baker J, Mankad K (2021). Rise in the incidence of abusive head trauma during the COVID-19 pandemic. Arch. Dis. Child..

[28] Kovler ML, Ziegfeld S, Ryan LM (2021). Increased proportion of physical child abuse injuries at a level I pediatric trauma center during the Covid-19 pandemic. Child Abus. Negl..

[29] Gilbert EH, Lowenstein SR, Koziol-McLain J, Barta DC, Steiner J (1996). Chart reviews in emergency medicine research: Where are the methods?. Ann. Emerg. Med..

