# Supplementary material for: Epidemiology and outcomes for level 1 and 2 traumas during the first wave of COVID19 in a Canadian centre
Source: Sci Rep. 2022 Nov 27;12:20345. doi: 10.1038/s41598-022-23625-8 (PMC9701679; doi:10.1038/s41598-022-23625-8)
Supplement: Supplementary file 2 — Supplementary Legends. [file 41598_2022_23625_MOESM2_ESM.docx]

Supplementary Figure 1: Saskatchewan Health Authority trauma triage guidelines flow chart for Saskatoon, SK. SBP: systolic blood pressure; RR: respiratory rate; GCS: Glascow Coma Score; ATV: all-terrain vehicle; MVC: motor vehicle collision; RUH: Royal University Hospital; JPCH: Jim Pattison Children’s Hospital; ETA: estimated time of arrival; ED: emergency department; MRP: most responsible physician; SFCC: system flow coordination center; TTL: trauma team leader.
