# Supplementary material for: Epidemiology and outcomes for level 1 and 2 traumas during the first wave of COVID19 in a Canadian centre
Source: Sci Rep. 2022 Nov 27;12:20345. doi: 10.1038/s41598-022-23625-8 (PMC9701679; doi:10.1038/s41598-022-23625-8)
Supplement: Supplementary file 3 — Supplementary Figure 1. [file 41598_2022_23625_MOESM3_ESM.pdf]

Trauma Triage Guidelines

Saskatchewan Health Authority

Pre-Hospital / Scene Call

Level I
(Any of the following)

Physiologic
- SBP < 90, SBP < 80 (age < 5), SBP < 70 (age < 1)
- RR < 10 or > 29 or requiring intubation
- GCS 12 or less with visible evidence of trauma OR clear history of significant trauma

Anatomic
- Penetrating Trauma to Head, Neck or Torso
- Severe Facial Injury with potential for airway compromise
- Suspected spinal CORD injury (neurological deficits)
- 2 or more long bone fractures
- Burns: 20% TBSA (2nd & 3rd Degree)

Mechanism
- Pedestrian or Cyclist vs. Car > 30 km/hr
- ATV or motorcycle vs. Car > 30km/hr
- Ejected from car
- Large Animal Trauma (non-MVC)
- Fall > 20 feet

Special population meeting Level II criteria, will be upgraded to Level I
- Pregnant patients > 20 weeks gestation
- Geriatrics (Age ≥ 65)

Level II
(Any of the following)

Mechanism
- Highway speed MVC
- Rollover
- Fall from 10-20 feet
- Death of same car occupant
- Rearward displacement of front axle
- Significant passenger compartment intrusion
- Other

Triage at RUH ED (in JPCH) to follow Activation Guidelines

Triage to Determine Level of Trauma

Level 1 **
Notify Trauma Team Lead

Level 2
Notify on shift ED Physician

**Notify Trauma team members

Paged out information:
- Age, Gender, location
- Mechanisms
- Vitals
- ETA

Intrafacility Transport*

Contact Trauma Team Lead (TTL) through SFCC

TTL determines level of trauma

Level 1 → **Notify Trauma team members

Level 2

TTL to discuss case with shift ED Physician at JPCH

Send to JPCH ED under accepting ED physician as Level 2 trauma

The ED Physician may upgrade any Level 2 to a Level 1 at their discretion at any point during the case of the patient or if the patient deteriorates on transport.

ED Physician must contact Trauma Team Lead with update and reason for upgrade. → **Notify Trauma team members

Trauma Consult

Send directly to Trauma Service under TTL

*** Includes patients being transported from St. Paul's Hospital**

**** ED Physician on shift is MRP until the Trauma Team Lead arrives and appropriate handover has occurred.**

- Patients who are found unresponsive with no history of trauma and no visible trauma will NOT be considered trauma patients. Simple falls while intoxicated do not qualify as significant trauma.
- If 3 or more Level II patients arrive at the same time, the ER Physician on shift may call the TTL to help manage these patients, at their discretion.
- If any doubt regarding activation, please contact TTL
